# Supplementary material for: The RNA-binding protein Squid regulates embryonic midgut development via Axin alternative splicing in Bombyx mori
Source: Commun Biol. 2026 Feb 10;9:449. doi: 10.1038/s42003-026-09692-x (PMC13022360; doi:10.1038/s42003-026-09692-x)
Supplement: Supplementary file 2 — Supplementary Fig. [file 42003_2026_9692_MOESM2_ESM.pdf]

Supplementary Table 1. Primers and probes used in this study

Primer	Forward (5’-3’)	Reverse Forward (5’-3’)
**Primers for qRT-PCR**		
BmAxin-L (896-1015)	GCAGCAGTCTCGATGGCGT	TGGCGGTGTCCTGCTCCTTA
BmAxin-L+S (115-335)	GTGTTCGCACAACCGCATTT	GCGAAGTAGAAGTTGAGTCGGTC
BmSmad4-1(1298-1429)	TCTTCGGCACAGAACAAACCACTA	TAGGTTGCGGTGCTGGACTG
BmSmad4 (1501-1684)	TGGACGGGTAACAACACGCTG	GTGTGTCAAGTTCAAAATACGCA
BmAtp5me	TCGCCTCTAATCAAGTTTGGACG	CGGATGACCTTCTCTTGGGCT
BmATP-PFK	GGCGATACCGTCCTTGGCAT	CGCAGCGATGTCACTCTTCC
BmHGD	CGGAATCAGAACGCCCAGTAT	GAAAACTCCGAACCAAAGCCC
BmHAGH	CCCTGTCATACAACTGGGCATA	AACCTGCCACATCCGCCTAA
BmIDH	GTCCAGCACAAGACTCCCGTT	CGAAGTCAATAGGAGCACCGA
BmPDHB	CACCCATCACAGAGGCAGGA	AGGTCTTGGCGGCTGAGTTT
Lipase member H-A	TTCTCACCAGTAATAGCGTCGGC	AAGGCGAGATGCGATTGGATA
BmFabp	ACATCAGTCATCGTCTGCCG	CAATAAACGCTTCCCGCACAA
BmApoltp	TGGATAAGCCCAATGGGGTTA	CGGGGGGTAGGAACAGGTATC
**Probes for *BmSquid* mutation screening**		
	TGGACGGGTAACAACACGCTG	GATAGCGGCACGCCATTT
***BmSquid* sgRNA sites**		
S1	GGAAATGCAGAGAACGGTGGCGG	

S2	CATAACAGTGCTGAGGCCCCAGG
S3	CCAAACACAGGCAGATCACGAGG

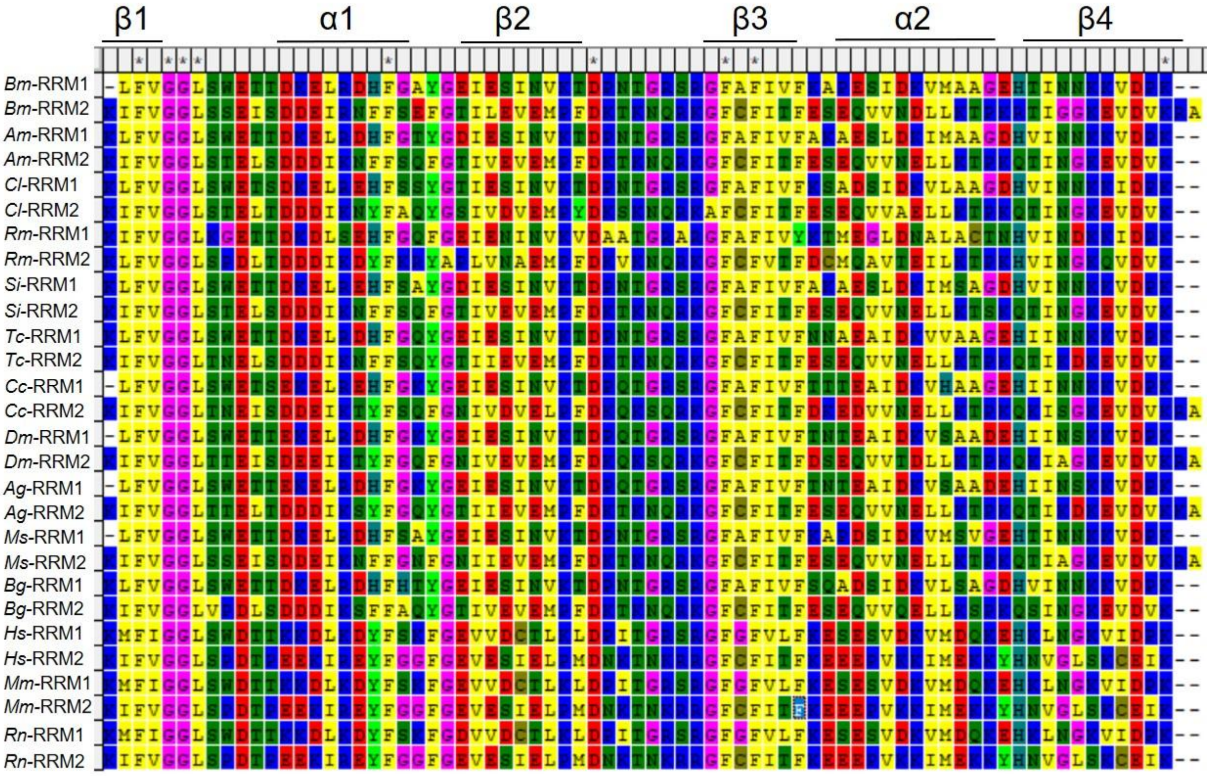

Supplementary Figure 1. Sequence alignment of Squid's RRMs across insect and mammal species. *Bm*, *Bombyx mori*. *Am*, *Apis mellifera*. *Cl*, *Cimex lectularius*. *Rm*, *Rhopalosiphum maidis*. *Si*, *Solenopsis Invicta*. *Tc*, *Tribolium castaneum*. *Cc*, *Ceratitis capitata*. *Dm*, *Drosophila melanogaster*. *Ag*, *Anoplophora glabripennis*. *Ms*, *Manduca sexta*. *Bg*, *Blattella germanica*. *Hs*, *Homo sapiens*. *Mm*, *Mus musculus*. *Rn*, *Rattus norvegicus*.

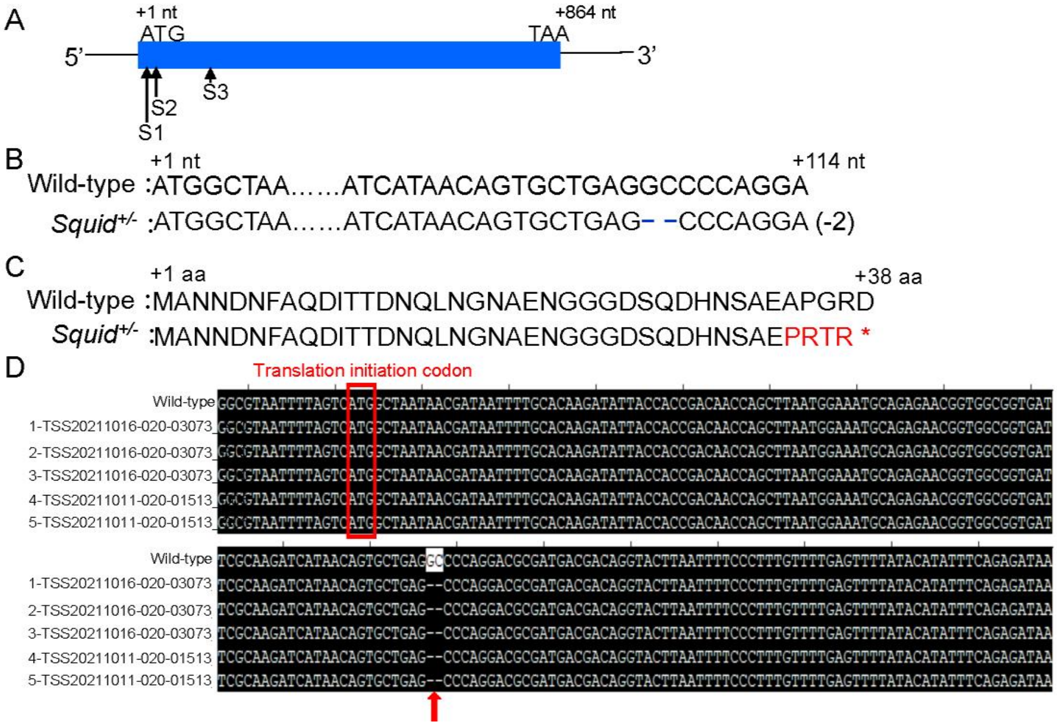

Supplementary Figure 2. CRISPR/Cas9-mediated knockout of *Squid* in silkworm. **A** Schematic diagram of sgRNA target sites. Blue box represents the exon of *Squid*, and black line denotes the gene locus. Three sgRNAs (S1, S2 and S3) were designed to target regions near the translation start site. **B** Nucleotide sequences of wild-type and *Squid*$^{-/-}$ mutant identified from heterozygous silkworms in heterozygous individual. Deleted bases are represented by blue dashes. **C** Predicted amino acid sequences of wild-type and *Squid*$^{-/-}$ mutant. A premature stop codon is marked with a red asterisk. **D** Sequence alignment of Squid genomic DNA from wild-type and homozygous mutant embryos. The translation initiation codon (ATG) is highlighted in a red box. Mutated sites are indicated with a red arrow.

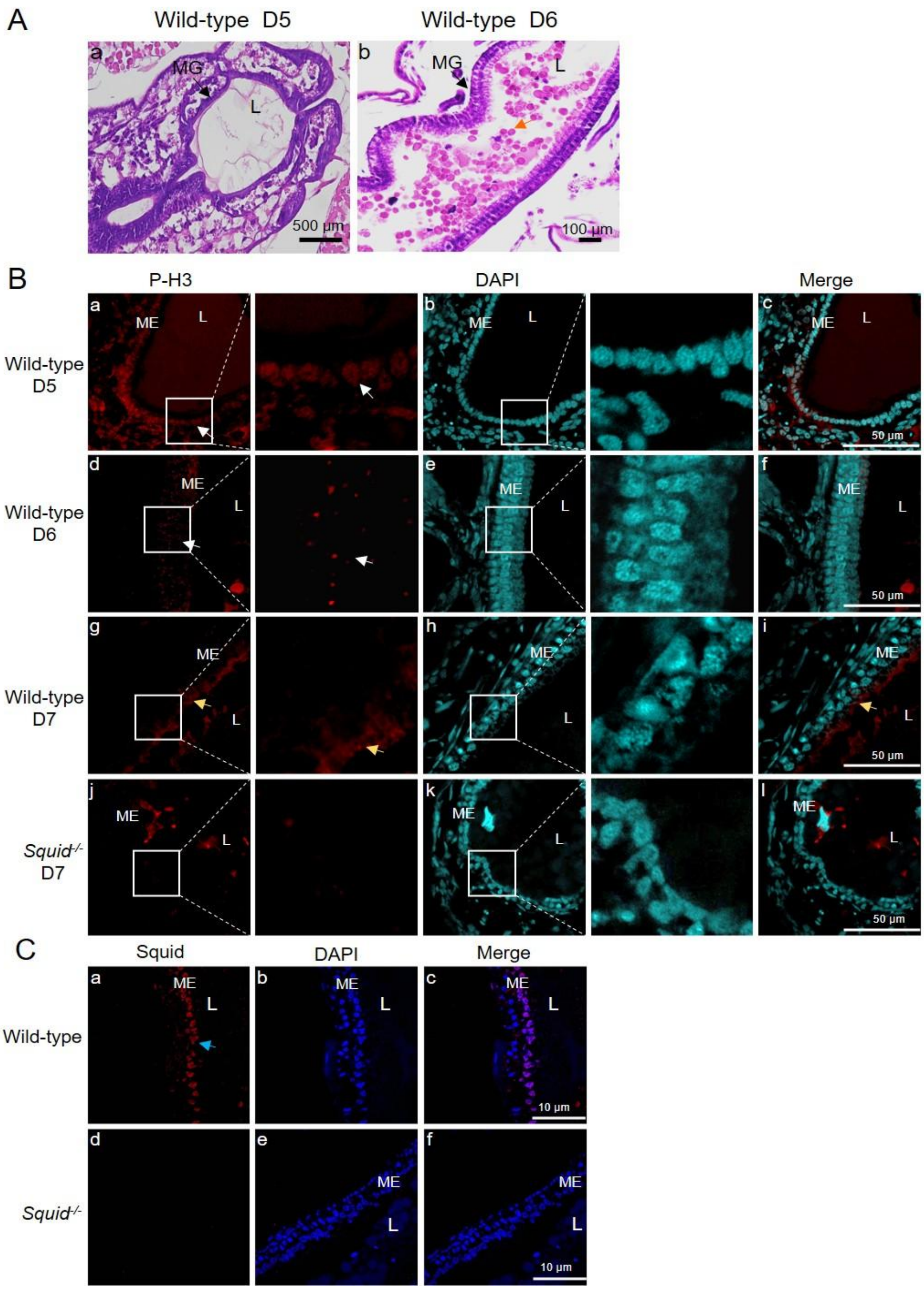

Supplementary Figure 3. Analysis of midgut morphology, cell proliferation, and Squid expression during embryogenesis. **A** Representative semi-thin cross-sections of the wild-type embryonic midgut at days 5 and 6 post-oviposition, showing tissue architecture. MG, midgut. Orange arrow point to lipid droplet-like vacuoles within the

lumen. The scale bar in a and b represents 500 µm and 100 µm, respectively. **B** Detection of cell proliferation signals in the midgut epithelium of wild-type from day 5 to day 7 and *Squid*$^{-/-}$ embryos on day 7. Proliferating cells are indicated by white arrows. The yellow arrows represent the non-specifically stained peritrophic matrix. The scale bars represent 50 µm. **C** Expression and localization of Squid protein. Squid signals are marked by blue arrow. ME, midgut epithelium. L, lumen. The scale bars represent 10 µm.

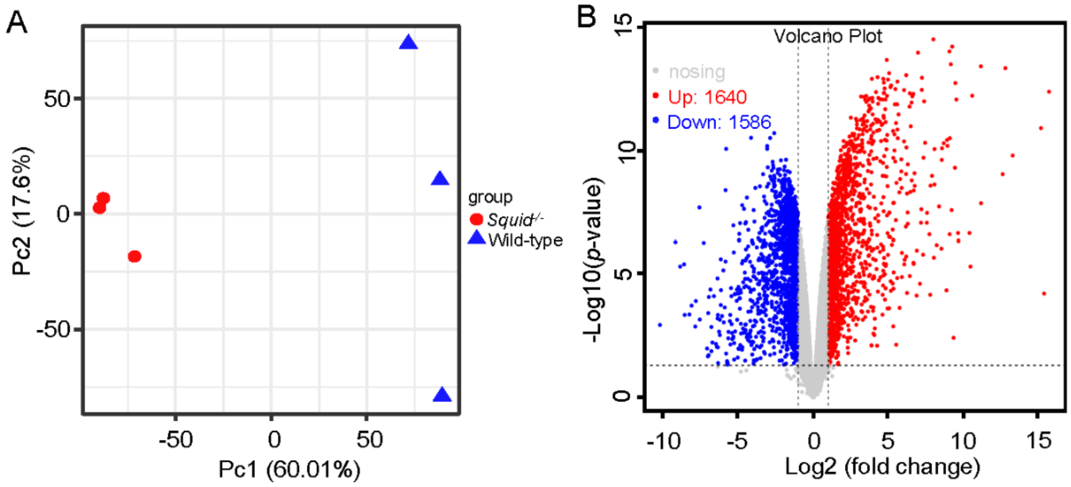

Supplementary Figure 4. Transcriptomic profiling of wild-type and *Squid*$^{-/-}$ embryos. **A** Principal component analysis (PCA) of RNA-seq data from wild-type and *Squid*$^{-/-}$ embryos. **B** Volcano plot showing differentially expressed transcripts (DETs). Red dots represent significantly up-regulated genes, and blue dots represent significantly down-regulated genes.

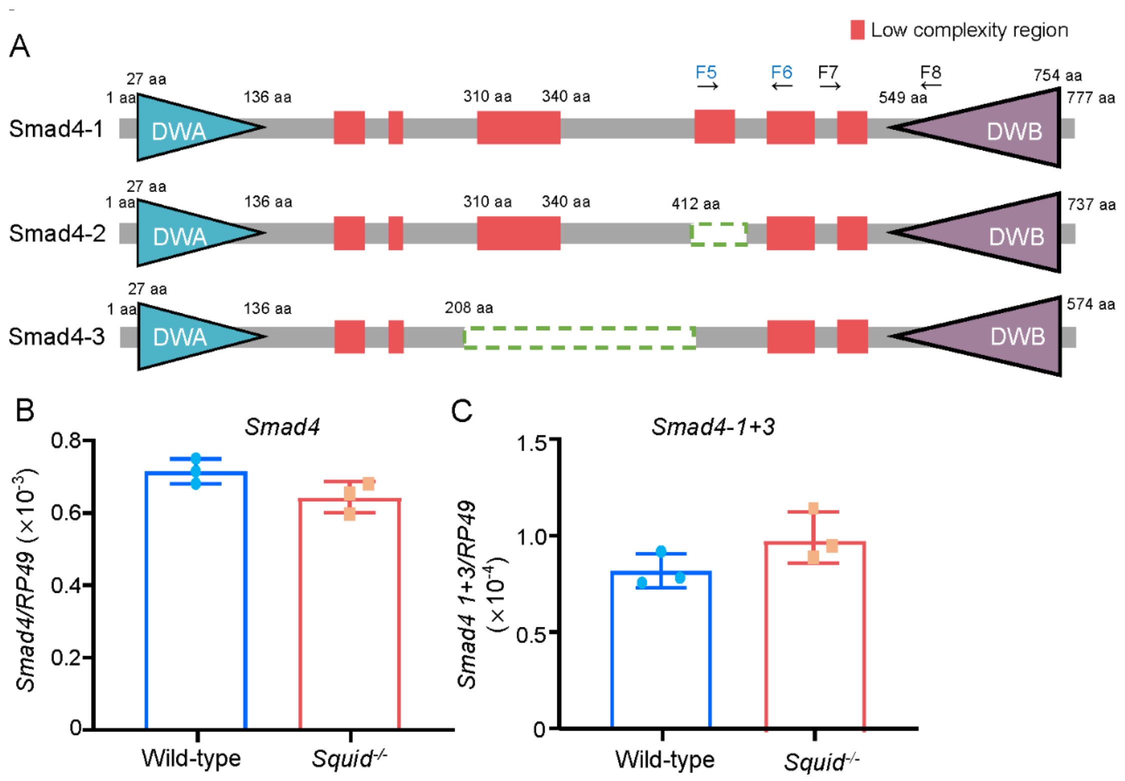

Supplementary Figure 5. Expression of *Smad4* isoforms in wild-type and *Squid*$^{-/-}$ embryos. A Schematic representation of *Smad4* splice variants Smad4-1, Smad4-2, and Smad4-3. The green dashed box indicates the region absent in Smad4-2 and Smad4-3 compared to Smad4-1. **B-C** RT-qPCR analysis of total *Smad4* mRNA (**B**) and the *Smad4-1+3* transcript subset (**C**) in wild-type and *Squid*$^{-/-}$ embryos. Primers F5 and F6 were used to amplify *Smad4-1+3* mRNA, while Primers F7 and F8 were used to amplify the shared region of the *Smad4-1*, *Smad4-2* and *Smad4-3* mRNA. The data are presented as mean ± SD. n=3 biologically independent samples.

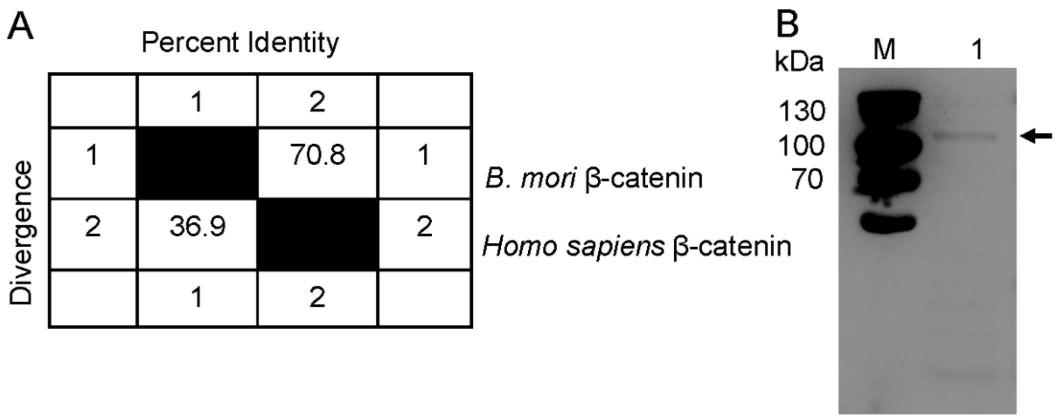

Supplementary Figure 6. Specificity validation of the anti-β-catenin antibody. **A** Sequence identity comparison of β-catenin between *B. mori* and *Homo sapiens*. **B** Western blot analysis of protein extracts from day 7 embryos using a commercial human anti-β-catenin antibody.

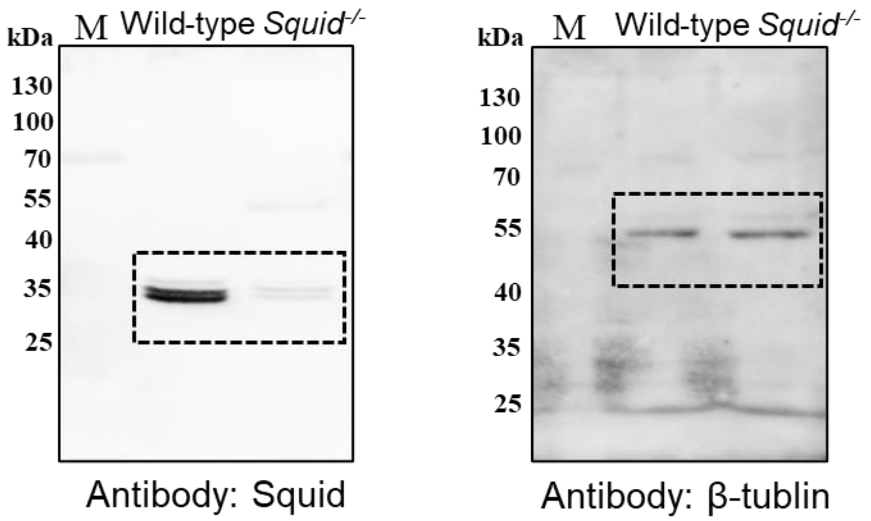

Supplementary Figure 7. Uncropped Western blot images corresponding to Fig. 1F. Western blot analysis of Squid and β-tublin expression in wild-type and *Squid*$^{-/-}$ embryos

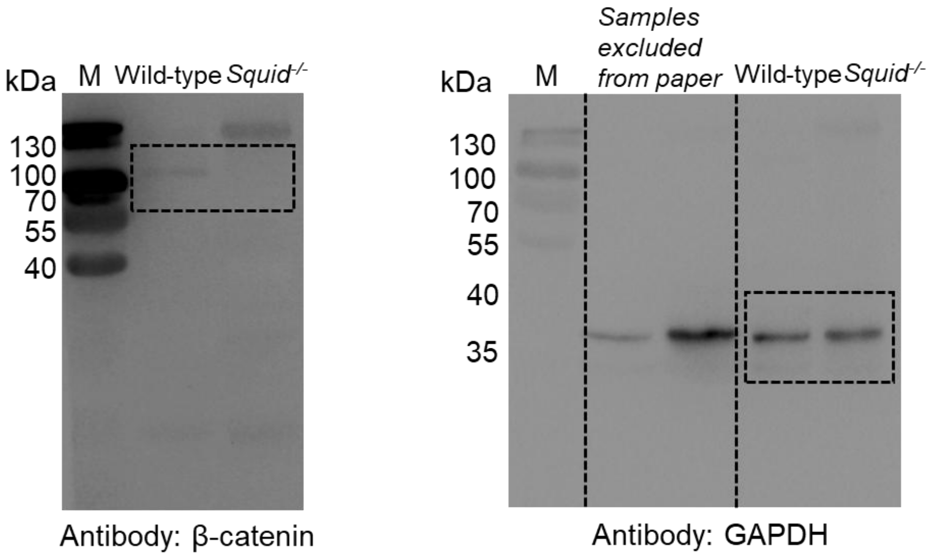

Supplementary Figure 8. Uncropped Western blot images corresponding to Fig.5D. Western blot analysis of β-catenin and GAPDH expression in wild-type and *Squid*$^{-/-}$ embryos
