# Supplementary material for: The RNA-binding protein Squid regulates embryonic midgut development via Axin alternative splicing in Bombyx mori
Source: Commun Biol. 2026 Feb 10;9:449. doi: 10.1038/s42003-026-09692-x (PMC13022360; doi:10.1038/s42003-026-09692-x)
Supplement: Supplementary file 3 — Description of Additional Supplementary File [file 42003_2026_9692_MOESM3_ESM.pdf]

Description of Additional Supplementary Files

File name: Supplementary Data

Description: The source data behind the graphs in the paper
