# Supplementary material for: The RNA-binding protein Squid regulates embryonic midgut development via Axin alternative splicing in Bombyx mori
Source: Commun Biol. 2026 Feb 10;9:449. doi: 10.1038/s42003-026-09692-x (PMC13022360; doi:10.1038/s42003-026-09692-x)
Supplement: Supplementary file 5 — Reporting Summary [file 42003_2026_9692_MOESM5_ESM.pdf]

nature portfolio

Corresponding author(s): Huimin Deng

Last updated by author(s): Jan 23, 2026

Reporting Summary

Nature Portfolio wishes to improve the reproducibility of the work that we publish. This form provides structure for consistency and transparency in reporting. For further information on Nature Portfolio policies, see our Editorial Policies and the Editorial Policy Checklist.

Statistics

For all statistical analyses, confirm that the following items are present in the figure legend, table legend, main text, or Methods section.

n/a	Confirmed	
☐	☒	The exact sample size (*n*) for each experimental group/condition, given as a discrete number and unit of measurement
☐	☒	A statement on whether measurements were taken from distinct samples or whether the same sample was measured repeatedly
☐	☒	The statistical test(s) used AND whether they are one- or two-sided<br>*Only common tests should be described solely by name; describe more complex techniques in the Methods section.*
☒	☐	A description of all covariates tested
☐	☒	A description of any assumptions or corrections, such as tests of normality and adjustment for multiple comparisons
☐	☒	A full description of the statistical parameters including central tendency (e.g. means) or other basic estimates (e.g. regression coefficient) AND variation (e.g. standard deviation) or associated estimates of uncertainty (e.g. confidence intervals)
☐	☒	For null hypothesis testing, the test statistic (e.g. *F*, *t*, *r*) with confidence intervals, effect sizes, degrees of freedom and *P* value noted<br>*Give P values as exact values whenever suitable.*
☒	☐	For Bayesian analysis, information on the choice of priors and Markov chain Monte Carlo settings
☒	☐	For hierarchical and complex designs, identification of the appropriate level for tests and full reporting of outcomes
☒	☐	Estimates of effect sizes (e.g. Cohen's *d*, Pearson's *r*), indicating how they were calculated

Our web collection on statistics for biologists contains articles on many of the points above.

Software and code

Policy information about availability of computer code

Data collection	Gene and protein sequences were downloaded from NCBI(https://www.ncbi.nlm.nih.gov/)
Data analysis	Graph Prism(Viewer mode) Software (version 8.0.2), Biovia Discovery Studio 2019 Client, AlphaFold3 (https://golgi.sandbox.google.com/), SWISS-MODEL (https://swissmodel.expasy.org/)

For manuscripts utilizing custom algorithms or software that are central to the research but not yet described in published literature, software must be made available to editors and reviewers. We strongly encourage code deposition in a community repository (e.g. GitHub). See the Nature Portfolio guidelines for submitting code & software for further information.

Data

Policy information about availability of data

All manuscripts must include a data availability statement. This statement should provide the following information, where applicable:

- Accession codes, unique identifiers, or web links for publicly available datasets
- A description of any restrictions on data availability
- For clinical datasets or third party data, please ensure that the statement adheres to our policy

All data required to evaluate the conclusions drawn in this paper are presented within the main text and the supplementary materials. The reads of transcriptome and RIP-Seq were deposited in the Sequence Read Archive (SRA) database of NCBI with the accession number SRP542796 and SRP543009, respectively. Additional data related to this study can be requested from the authors.

Research involving human participants, their data, or biological material

Policy information about studies with human participants or human data. See also policy information about sex, gender (identity/presentation), and sexual orientation and race, ethnicity and racism.

Reporting on sex and gender	Not applicable.
Reporting on race, ethnicity, or other socially relevant groupings	Not applicable.
Population characteristics	Not applicable.
Recruitment	Not applicable.
Ethics oversight	Not applicable.

Note that full information on the approval of the study protocol must also be provided in the manuscript.

Field-specific reporting

Please select the one below that is the best fit for your research. If you are not sure, read the appropriate sections before making your selection.

☒ Life sciences ☐ Behavioural & social sciences ☐ Ecological, evolutionary & environmental sciences

For a reference copy of the document with all sections, see nature.com/documents/nr-reporting-summary-flat.pdf

Life sciences study design

All studies must disclose on these points even when the disclosure is negative.

Sample size	We performed the power analysis to predetermine sample size.
Data exclusions	We did not exclude the data.
Replication	All data included in the study are reproducible.
Randomization	The animals were allocated into experimental group in random
Blinding	The investigators were blinded to group allocation during data collection and analysis.

Reporting for specific materials, systems and methods

We require information from authors about some types of materials, experimental systems and methods used in many studies. Here, indicate whether each material, system or method listed is relevant to your study. If you are not sure if a list item applies to your research, read the appropriate section before selecting a response.

Materials & experimental systems

n/a	Involved in the study	
☐	☒	Antibodies
☐	☒	Eukaryotic cell lines
☒	☐	Palaeontology and archaeology
☐	☒	Animals and other organisms
☒	☐	Clinical data
☒	☐	Dual use research of concern
☒	☐	Plants

Methods

n/a	Involved in the study	
☒	☐	ChIP-seq
☒	☐	Flow cytometry
☒	☐	MRI-based neuroimaging

Antibodies

Antibodies used	The antibodies used in the present study were: anti-Squid antibody (Prepared by our laboratory), anti-β-catenin antibody (Immunoway, YM3403), anti-GADPH antibody (Proteintech, 60004-1-Ig), anti-β-tublin (Dingguo biology, TB002-R), Histone H3 (Phospho Ser10, P-H3)(Immunoway, YP0129), Alexa Fluor 594(Invitrogen, USA).

Validation	Each antibodies was verified by western blot and immunohistochemistry experiment, and relevant information is presented in the main text and and the supplementary materials.

Eukaryotic cell lines

Policy information about cell lines and Sex and Gender in Research

Cell line source(s)	Bombyx mori BmN cell line was obtained from lab preservation.
Authentication	Bombyx mori BmN cell line was not authenticated.
Mycoplasma contamination	Not tested for Mycoplasma contamination
Commonly misidentified lines (See ICLAC register)	None.

Animals and other research organisms

Policy information about studies involving animals; ARRIVE guidelines recommended for reporting animal research, and Sex and Gender in Research

Laboratory animals	Bombyx mori P50, the age of Bombyx mori used in each experiment is described in detail in the manuscript
Wild animals	No wild animals were used in this study
Reporting on sex	This manuscript does not report on sex
Field-collected samples	No field collected samples were used in this study
Ethics oversight	All animal experiments complied with the management and use policies of South China Normal University

Note that full information on the approval of the study protocol must also be provided in the manuscript.

Plants

Seed stocks	Not applicable.
Novel plant genotypes	Not applicable.
Authentication	Not applicable.
